# Can words heal? Using affect labeling to reduce the effects of unpleasant cues on symptom reporting

**DOI:** 10.3389/fpsyg.2014.00807

**Published:** 2014-07-22

**Authors:** Elena Constantinou, Maaike Van Den Houte, Katleen Bogaerts, Ilse Van Diest, Omer Van den Bergh

**Affiliations:** Health Psychology Group, Department of Psychology, University of LeuvenLeuven, Belgium

**Keywords:** emotion regulation, affect labeling, symptom reporting, self-reported affect, general inhibitory capacity

## Abstract

Processing unpleasant affective cues induces elevated momentary symptom reports, especially in persons with high levels of symptom reporting in daily life. The present study aimed to examine whether applying an emotion regulation strategy, i.e. affect labeling, can inhibit these emotion influences on symptom reporting. Student participants (*N* = 61) with varying levels of habitual symptom reporting completed six picture viewing trials of homogeneous valence (three pleasant, three unpleasant) under three conditions: merely viewing, emotional labeling, or content (non-emotional) labeling. Affect ratings and symptom reports were collected after each trial. Participants completed a motor inhibition task and self-control questionnaires as indices of their inhibitory capacities. Heart rate variability was also measured. Labeling, either emotional or non-emotional, significantly reduced experienced affect, as well as the elevated symptoms reports observed after unpleasant picture viewing. These labeling effects became more pronounced with increasing levels of habitual symptom reporting, suggesting a moderating role of the latter variable, but did not correlate with any index of general inhibitory capacity. Our findings suggest that using an emotion regulation strategy, such as labeling emotional stimuli, can reverse the effects of unpleasant stimuli on symptom reporting and that such strategies can be especially beneficial for individuals suffering from medically unexplained physical symptoms.

## INTRODUCTION

The perception of signals coming from the body (interoception; Cameron, 2001) has been strongly linked to emotional processes. Emotion theories from James (1884) to newer views (Damasio, 1994; Wiens, 2005) consider bodily signals as essential elements of emotional experiences, while theories of interoception emphasize the role of an affective component of bodily sensations in the perception of body state (Craig, 2003). This inter-connection is further supported by neurobiological findings showing intertwined neural pathways for the representation of emotional experiences and the perception of bodily sensations (Craig, 2009).

This link between emotion and interoception has also been observed at the behavioral level, as emotional states seem to interfere with the perception of bodily signals, a process with important implications for the subjective experience of physical symptoms. Research has shown for example that the presence of unpleasant cues augments the perception of experimentally induced bodily sensations, like pain (de Wied and Verbaten, 2001; Meagher et al., 2001), dyspnea (Von Leupoldt et al., 2006), or esophageal stimulation (Phillips et al., 2003), as well as the reporting of physical symptoms in general (Salovey and Birnbaum, 1989). Recent work has further shown that unpleasant cues can result in increased symptom reports even without any physiological challenge, although this effect seems to be mostly observed in persons reporting frequent bodily complaints in daily life not explained by organic dysfunction (high habitual symptom reporters; Bogaerts et al., 2010; Constantinou et al., 2013). Such differential effects of unpleasant cues have been also reported for patients with functional syndromes (Montoya et al., 2005) or people scoring high on trait Negative Affectivity (Bogaerts et al., 2005).

Thus, it seems that some people are more prone to be influenced by emotional information than others in their subjective experience of current body state. Interestingly, both people high on trait Negative Affectivity (Gross and John, 2003; Moberly and Watkins, 2008) and patients with functional syndromes (Waller and Scheidt, 2006; van Middendorp et al., 2008) have been found to use ineffective strategies to regulate their emotion, like suppression and avoidance, while less effort for emotion regulation has been linked to increased symptom reporting during periods of stress in non-clinical samples (Goldman et al., 1996). Furthermore, brain imaging studies have shown that patients with functional syndromes show greater activation in limbic networks and reduced activation in prefrontal inhibitory systems compared to controls during unpleasant bodily stimulation (Mayer et al., 2005; Elsenbruch et al., 2010; Tillisch et al., 2011). These findings overall suggest that individuals who tend to over-report symptoms exhibit deficits in emotion regulation, and these deficits may contribute to the “fusion” of emotional experiences with the symptom perception process. Based on this notion, it can be hypothesized that enforcing emotion regulatory processes may reduce affective influences on symptom reporting in these groups, a proposition that is examined in this paper.

Emotion regulatory processes, that is “the processes by which individuals influence which emotions they have, when they have them and how they experience and express these emotions” (Gross, 1998; p. 275) include strategies employed intentionally to down-regulate emotion (explicit emotion regulation), as well as processes that incidentally result in down-regulation (implicit emotion regulation; Gyurak et al., 2011). Although, explicit emotion regulation strategies, like cognitive reappraisal or behavioral suppression, have dominated emotion regulation research (Berkman and Lieberman, 2009; Gyurak et al., 2011), recent work has focused on the effects of incidental emotion regulation strategies, such as affect labeling. The latter can be seen as an operationalization of the commonsensical notion that verbalizing feelings can dampen them (Hariri et al., 2000). Affect labeling tasks typically include presentations of emotional stimuli, but instead of explicit instructions to down-regulate emotions, participants are asked to assign emotional labels to the stimuli.

Assigning emotional labels has been found to reduce amygdala activation and increase inhibitory activation at prefrontal areas compared to non-verbally matching target faces with similar in expression facial stimuli (Hariri et al., 2000), to labeling non-emotional features of target stimuli (Lane et al., 1997; Lieberman et al., 2007) and to merely viewing pictorial cues (Taylor et al., 2003). Thus, affect labeling seems to activate inhibitory processes and exert emotion regulatory benefits (Lieberman et al., 2007) in a way similar to explicit emotion regulation (Ochsner et al., 2004). These benefits also extend to self-reported affect (Lieberman et al., 2011) and autonomic reactivity, with studies indicating reduced physiological responding following labeling of emotional pictures (McRae et al., 2009). The attenuating effects of labeling seem to persist in time (Tabibnia et al., 2008), while recent data support its usefulness in the context of exposure therapy (Kircanski et al., 2012).

A strategy like affect labeling could be especially beneficial for persons experiencing medically unexplained physical symptoms, as they typically tend to avoid emotional experiences (van Middendorp et al., 2008) and are characterized by a difficulty in recognizing and expressing emotions (alexithymia; De Gucht and Heiser, 2003; Waller and Scheidt, 2006). Labeling an emotion implies the activation of prior conceptual knowledge about emotion categories, which among others includes ways to act upon specific emotions (Barrett, 2006). Thus, an affect labeling procedure may initiate emotion regulatory processes, that otherwise would not spontaneously occur in people who tend to over-report symptoms, by engaging them in categorizing affect. This is assumed to reduce experienced negative affect, which in turn may lead to a reduction in symptom reporting.

A secondary question is whether these hypothesized attenuating effects of affect labeling on symptom reports relate to dispositional regulatory capacities of high symptom reporters. Prior research suggests that successful emotion regulation depends on executive functioning abilities (Hofmann et al., 2012), and shares common neural substrates related to inhibitory processes, like the right VLPFC, with other forms of self-control (Cohen and Lieberman, 2011). As high symptom reporters seem to also perform poorly in tasks assessing executive control, like motor inhibition tasks (Glass et al., 2011), we aimed to examine whether labeling effects on symptom reporting depend on individual differences in general inhibitory abilities.

Besides behavioral tasks, an additional measure of regulatory capacity is used in this study, namely heart rate variability (HRV). HRV is considered a physiological marker of emotion regulation (Thayer and Lane, 2000; Appelhans and Luecken, 2006) as it reflects the capacity of efferent signals to modulate cardiac activity according to situational demands. It has been associated to self-reported (Fabes and Eisenberg, 1997) and spontaneous regulation of emotion under unpleasant contexts (Pu et al., 2010).

To sum up, the present study aimed to examine: (a) whether affect labeling can reverse the augmenting effects of unpleasant cues on symptom reporting in a non-clinical sample, (b) whether this possible reduction is modulated by the level of habitual symptom reporting, (c) whether such a reduction is predicted by changes in experienced affect and d) whether it correlates with behavioral, self-reported and physiological indices of self-regulation.

To this end, students with varying levels of habitual symptom reporting completed a modified Affect Labeling task, which included viewing pleasant and unpleasant pictures under three conditions: merely viewing, labeling the emotion of the picture, and labeling the content of the picture. Picture viewing was followed by affect ratings and a symptom checklist. Participants also completed a computerized motor inhibition task (Parametric Go-No Go task) and an HRV assessment. We expected that: (a) labeling the emotion of the pictures will lead to reduced self-reported affect, as well as reduced symptom reports after unpleasant pictures compared to merely viewing the pictures and non-emotional labeling, (b) the effects of emotional labeling on symptom reports will be more pronounced at higher levels of habitual symptom reporting, (c) the reduction in symptom reports during labeling will be predicted by reductions in affect ratings, and (d) this reduction in symptom reports will correlate positively with inhibitory capacity as assessed by the Go-No Go task, with self-reported self-regulation capacity and with HRV indices.

## MATERIALS AND METHODS

### SAMPLE

Data were collected from 63 healthy first year psychology students (seven male, *M*_age_ = 19.02, SD_age_ = 1.52), who were invited based on their scores on the Checklist for Symptoms in Daily Life (CSD; Wientjes and Grossman, 1994) obtained during collective psychological testing. Participants with scores across the whole spectrum of habitual symptom reporting were selected for participation. Specifically, the initial pool of students (*N* = 401) was divided into four groups based on the quartiles of the total score of the CSD (scores could range from 39 to 195) and an equal amount of participants was invited from each group. Due to unbalanced response rates, an equal number of participants from each group was not feasible: the CSD scores of the final sample followed a rather normal distribution (range = 50–116, *M* = 82.87, SD = 15.20).

Participants were excluded if they reported to (a) have a diagnosis of a medical or psychiatric disorder, (b) have an electronic implant (e.g., pacemaker) and (c) use anxiolytic medication, antidepressants, or beta-blockers. The data for the Affect Labeling task of two participants who failed to appropriately follow the instructions were excluded from analyses. Students were compensated for their participation with course credit or a small monetary reward. The study was approved by the Multidisciplinary Ethical Committee of the Faculty of Psychology and Educational Sciences of the University of Leuven.

### TASKS

#### Modified affect labeling task

The typical Affect Labeling task (Lieberman et al., 2007) was modified as follows: (a) instead of faces, emotional stimuli consisted of pictures selected from the International Affective Picture System (IAPS; Lang et al., 2005), (b) stimuli were grouped into sets homogenous in valence, and (c) a symptom reporting phase was added to each trial. Emotional pictures were selected using valence and arousal ratings of IAPS pictures provided by Belgian participants in other studies. They were grouped into six sets of 10 pictures, three pleasant and three unpleasant^1^, so that sets of similar valence did not differ from each other in valence or arousal^2^. Furthermore, in each pleasant set, five pictures were scoring high on excitement (e.g., skiing) and five on contentment (e.g., cute animals) based on Mikels et al. (2005) norms, while in each unpleasant set five pictures were high on sadness (e.g., cemetery) and five on fear (e.g., gun).

The task consisted of six picture viewing trials, three with pleasant and three with unpleasant pictures. During each trial, 10 pictures were presented in the upper part of the screen for 6 s each (no inter-stimulus interval) and participants had to perform one of three tasks: a) VIEW: merely watch the pictures, (b) LABEL EMOTION: select from two emotion words presented under the picture (two out of: *content*, *excited*, *sad*, *afraid)* the one most applicable to the depicted emotion, and (c) LABEL CONTENT: select from two words presented under the picture (two out of: *object*, *animal*, *human*, *landscape)* the one most applicable to describe the content of the picture.

Each trial consisted of: (a) a 3-s presentation of a word cue signaling the task participants had to do (VIEW, LABEL EMOTION, LABEL CONTENT), (b) a 60-s period of picture viewing, and (c) a 90-s inter-trial period, during which participants completed electronically affect ratings and a symptom checklist (see further).

#### Motor inhibition task

Motor inhibition was assessed with the Parametric Go-No Go task (PGNG; Langenecker et al., 2007), a reaction-time task with increasing inhibitory demands. During the PGNG, participants see letters on a computer screen (black small case letters on a white background) presented one after the other for 500 ms each without inter-stimulus interval. At the first level of the task, participants press a button whenever one of three target letters (*x*, *y*, *z*) appears on screen as soon as possible. At the second level, participants are asked to press a button every time one of two targets (*x* or *y*) appears on screen, but only when the current target is different from the previous one, i.e., respond to a “*y*,” after responding to an “*x*” (Go trials). They must inhibit their response when the current target is the same as the previous one, i.e., inhibit responding to an “*x*” after responding to an “*x*” (No Go trials). The third level is identical to the second but with three targets this time (*x*, *y*, *z*). The accuracy (percentage of correct responses) at the “No-Go” trials of the last two levels was used in analyses as an index of behavioral inhibition capacity.

### MEASURES

#### Heart rate variability

Baseline heart rate was recorded using a Polar RS800CX watch (Polar Electro Oy, Kempele, Finland), with a chest strap on which electrolyte gel was applied, placed just below the chest. Participants were asked to sit in a comfortable chair, relax, and breathe normally for 5 min. The experimenter holding the watch was seated at the side of the participant, so that the watch was not visible. Polar watches are commonly used to collect heart rate and they have been found to provide data comparable to those by traditional ECG electrodes (Nunan et al., 2009). The recorded *R*-*R* intervals were off-line processed with the ARTiiFACT software (Kaufmann et al., 2011) to extract HRV parameters by two independent raters (inter-rater reliability: *r* = 0.92–0.99). For the purposes of this study, the RMSSD time-domain parameter and the High Frequency (HF) frequency-domain parameter were used.

Due to technical problems, the data of eleven participants were not used, while another participant was excluded due to smoking right before the recording.

#### Habitual symptom reporting

The CSD based on Wientjes and Grossman’s symptom checklist (1994) was used to assess participants’ tendency for symptom reporting in everyday life. In this 39-item questionnaire participants rated on a 5-point Likert Scale (1 = never, 5 = very often) the extent to which they experienced a variety of symptoms, e.g., headache, dizziness, back pain, etc. over the past year. Total scores (39–195) were used for the selection of participants. The reliability (Cronbach’s alpha) of the total scores exceeded 0.90 in our sample.

#### Self-control

As a self-report measure of self-regulation, the Dutch version of the Brief Self-Control Scale (Tangney et al., 2004) was used. This 13-item questionnaire consists of statements like “I am good at resisting temptation” and participants have to rate the extent each statement reflects how they are on a 5-point Likert scale (1 = not at all, 5 = very much). Internal consistency and test–retest reliability has been found to exceed the criterion of 0.70 in the English (Tangney et al., 2004) and the Dutch version (as reported in Finkenauer et al., 2005).

#### Emotion regulation

The Dutch version of the 10-item Emotion Regulation Questionnaire (Gross and John, 2003) was used to assess people’s reliance on cognitive reappraisal to regulate their emotions (six items; e.g.,: “I control my emotions by changing how I think about the situating I’m in”) or suppression (four items; e.g.,: “I control my emotions by not expressing them”). Participants rated their level of agreement with each statement on a 7-point Likert scale. A reappraisal and a suppression score were calculated.

#### State symptom reports

A list of 14 complaints was incorporated in the modified Affect Labeling task after each trial. The list included 10 everyday symptoms previously used in a similar picture viewing paradigm (Bogaerts et al., 2010; Constantinou et al., 2013) and 4 additional gastro-intestinal symptoms added for exploratory reasons (*chest tightness*, *pounding of the heart*, *headache*, *fatigue*, *not able to breathe deeply*, *rapid heartbeat*, *dizziness*, *muscular pain*, *stomach or abdominal cramps*, *nausea*, *stomach pain*, *bloated stomach*, *reflux sensations*, *burning feeling in the eyes*). Participants rated the presence of each of these complaints during picture viewing on a 5-point Likert scale (1 = not at all, 5 = very strong). A total symptom score (ranging from 14 to 70) was computed for each trial.

#### Affect ratings

After each trial of the modified Affect Labeling task, participants also rated their experienced affect during picture viewing in the dimensions of valence, arousal and control using a computerized nine-point version of the Self-assessment Manikin (SAM; Bradley and Lang, 1994). With this pictorial scale, values for each of the three dimensions are represented by nine human figures depicting gradually increasing valence, arousal or control, and participants respond by choosing the appropriate figure.

### PROCEDURE

Participants selected based on their CSD scores were invited by e-mail to participate in a study about “emotions and reaction time.” For the HRV assessment, participants were asked to refrain from alcohol for 12 h and smoking, physical exercise and caffeine for 4 h before the experiment. They were also asked not to eat 2 h prior to the experiment.

Upon arrival, participants gave written informed consent and their compliance with the aforementioned instructions was assessed. A series of factors that may influence HRV was also recorded: smoking frequency, alcohol and caffeine consumption, exercise and BMI. They also completed a first set of questionnaires (General Health Questionnaire and the CSD). After questionnaire completion, the equipment was attached and the HRV recording took place. Afterwards, participants were seated in front of a desktop computer and completed the PGNG task and the Affect Labeling task in counterbalanced order.

The three levels of the PGNG task were completed at fixed order while for the Affect Labeling task the six picture viewing trials were presented in a semi-counterbalanced order. Specifically, 12 different orders were constructed, so that each of the six trials was presented twice in a specific order position, while making sure that each of the three sets of pleasant/unpleasant pictures was presented four times for each of the three tasks (View, Label Emotion, Label Content).

A 10-min break was added between the PGNG and the labeling task to avoid fatigue effects, during which participants completed the rest of questionnaires (Self-Control Questionnaire, Emotion Regulation Questionnaire). The researcher was present in the room throughout the experiment and seemed to be working at the other side of the room, so that participants did not feel disturbed or watched, but could ask questions whenever needed.

The Affect 4.0. software (Spruyt et al., 2010) was used for stimuli presentation and timing of the labeling task, while E-prime 1.0 (Schneider et al., 2002) was used for the presentation of the PGNG task.

### DATA ANALYSES

Data from the Affect Labeling task were analyzed with Repeated Measures ANCOVA with Emotion (positive/negative) and Task (View/Label Content/Label Emotion) as within variables and CSD scores as a continuous predictor (after centering to the mean). Repeated measures ANCOVAs were run with the affect ratings after each trial (valence, arousal and control) as dependent variables to confirm the emotion regulatory effects of the task and with the total symptom scores to assess the effects of emotion regulation on symptom reporting. Greenhouse–Geisser corrected *p*-values and epsilon are reported when the sphericity assumption was violated, while follow-up comparisons were examined with *post hoc* Bonferroni tests. Interactions involving the continuous predictor were inspected by plotting effects at different levels of the continuous predictor (average, +1 SD, -1 SD).

To test the relationship among labeling effects and other regulatory measures, Pearson’s bivariate correlations were conducted among the accuracy level of PGNG, HRV indices, the questionnaires and the emotion and content labeling effects on symptom reports. The latter variables were calculated by subtracting the total symptom scores during the viewing trial from those of the emotion labeling trial and the content labeling trial, respectively. Similar difference scores were calculated for valence and arousal ratings after each trial to examine whether labeling effects on emotion predict labeling effects on symptom reports in multiple regressions. All analyses were conducted with STATISTICA 11.0 software (Statsoft, Inc., Tulsa, OK, USA).

## RESULTS

### SAMPLE CHARACTERISTICS

Sample characteristics are illustrated at **Table 1**. All participants were healthy and did not take medication, except for one participant who had medication controlled asthma. The vast majority were non-smokers, while about 81% of the sample did not consume coffee regularly and 89% consumed alcohol rarely or weekly. Most female participants were using contraceptive pills.

**Table 1:** Means and SDs on descriptive variables, questionnaires, PGNG, and HRV indices.

	*N*	Mean	SD	Minimum	Maximum
Age	63	19.02	1.52	17.00	26.00
BMI	63	21.82	2.81	16.44	29.74
Last meal (h)	63	3.31	2.58	0.25	14.00
**Questionnaires**
CSD total	63	82.87	15.20	50	116
SCQ total	63	40.1	7.44	24	54
ERQ-reappraisal	63	25.78	6.01	8	37
ERQ-suppression	63	13.44	5.2	4	27
**HRV indices**
RMSSD	51	52.68	21.83	19.59	110.61
HF abs. (ms^2^)	51	1325.21	1232.26	135.82	5626.69
HF nu	51	49.05	17.18	17.58	82.25
**PGNG task**
Level 2-accuracy %	61	81.31	15.19	33.33	100.00
Level 3-accuracy %	62	62.10	15.50	28.86	92.86

*CSD, Checklist for Symptoms in Daily Life; SCQ, Self-Control Questionnaire; ERQ, Emotion Regulation Questionnaire; HRV, Heart rate variability; RMSSD, root mean square of successive differences; HF abs. (ms^2^), High Frequency absolute values of power; HF nu, high frequency normalized units; PGNG, parametric go-no go.*

### MANIPULATION CHECKS

To examine whether the Affect Labeling task was successful in regulating affect, repeated measures ANCOVAs were conducted with valence, arousal and control ratings as dependent measures (see **Table 2** for means and SDs).

**Table 2:** Means and SDs for all dependent variables of the Affect Labeling task (*N* = 61).

Measure	Trial					
	Positive			Negative		
	View	Emotion label	Content label	View	Emotion label	Content label
Valence (1–9)	7.88 (1.07)	7.66 (1.08)	7.36 (1.35)	2.46 (1.12)	3.25 (1.48)	3.38 (1.53)
Arousal (1–9)	3.46 (2.28)	3.79 (2.11)	3.36 (1.71)	5.02 (1.75)	4.43 (1.79)	4.33 (1.68)
Control (1–9)	6.66 (1.64)	6.44 (1.64)	6.25 (1.63)	3.39 (2.15)	3.82 (2.11)	4.13 (1.84)
Symptoms (14–70)	14.98 (1.52)	14.87 (1.28)	14.77 (1.02)	17.20 (2.84)	16.08 (2.21)	16.34 (2.23)

**FIGURE 1:**
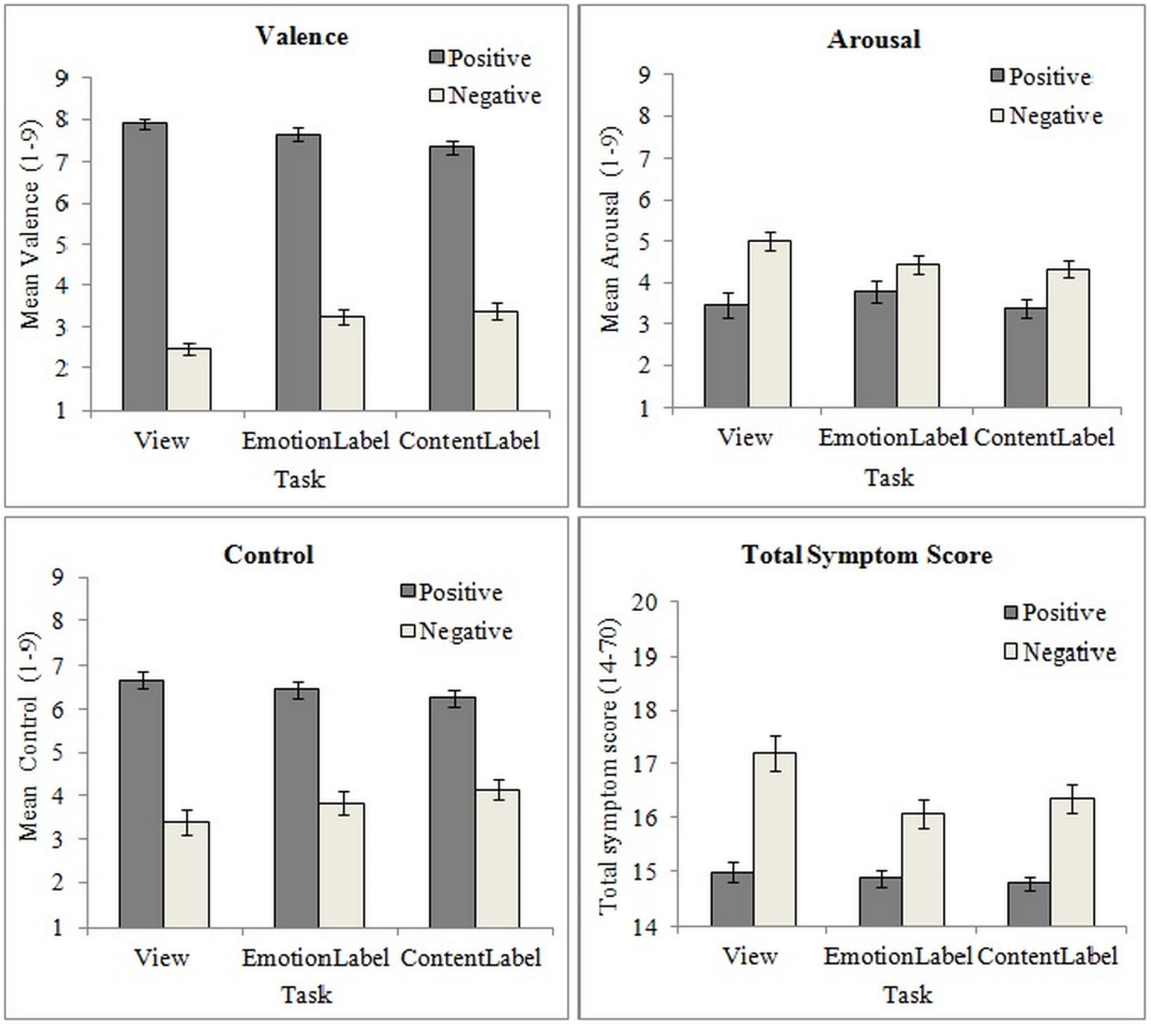
**Emotion × Task interaction effect for valence (top left), arousal (top right) and control ratings (bottom left) and symptom reports (bottom right) after each trial**.

**FIGURE 2:**
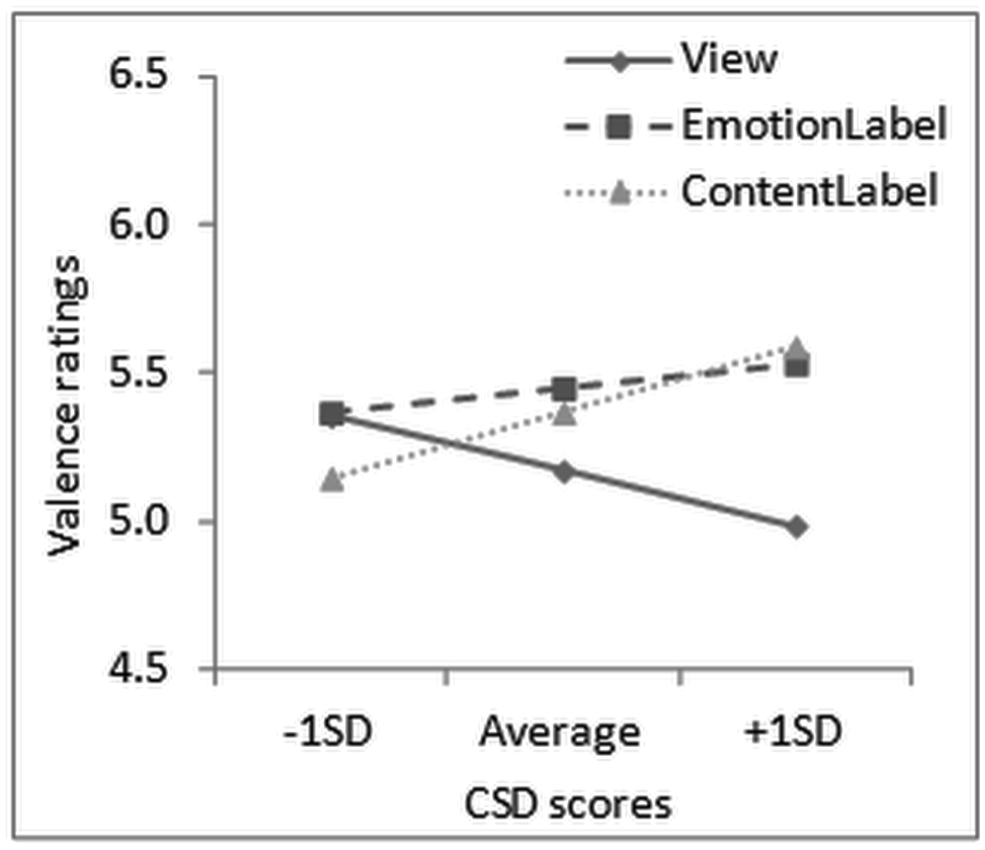
**Interaction of Task with habitual symptom reporting scores for valence ratings after each trial of the Affect Labeling task.** CSD = Checklist for Symptoms in Daily Life.

For valence, an Emotion main effect, *F*(1,59) = 753.81, *p* < 0.001, partial η^2^ = 0.93, and an Emotion × Task interaction, *F*(2,118) = 15.53, *p* < 0.001, partial η^2^ = 0.21 were found, indicating that negative trials led to significantly more unpleasantness than positive ones, but within the negative trials, both labeling conditions were rated as less unpleasant (emotion labeling: *M*_diff_ = -0.79, 95% CI [-1.28, -0.30], *p* < 0.001, content labeling: *M*_diff_ = -0.92, 95% CI [-1.41, -0.43], *p* < 0.001) compared to merely viewing unpleasant pictures (**Figure 1**). For the positive trials, the opposite effect was observed, with labeling conditions rated as more unpleasant compared to merely viewing pleasant pictures, especially Content Labeling (*M*_diff_ = 0.53, 95% CI [0.13, 0.92], *p* < 0.01). Furthermore, Task interacted significantly with CSD scores (the continuous predictor), *F*(2,118) = 5.17, *p* < 0.01, partial η^2^ = 0.08. This interaction was further explored with separate analyses for each task, which showed that the CSD scores had a nearly significant effect on valence ratings for merely viewing, *F*(1,53) = 3.49, *p* = 0.07, partial η^2^ = 0.06, but not for the two labeling conditions. Plotting the effect of Task at different levels of CSD scores (average, +1 SD, -1 SD), indicated that the difference between viewing and the two labeling conditions was more pronounced as CSD scores increased (**Figure 2**).

As for arousal ratings, main effects of Emotion, *F*(1,59) = 21.33, *p* < 0.001, partial η^2^ = 0.27, and Task, *F*(2,118) = 3.66, *p* < 0.05, partial η^2^ = 0.06, showed that overall negative trials were rated as more arousing than positive trials, while Content Labeling led to reduced perceived arousal compared to merely viewing pictures, *M*_diff_ = 0.39, 95% CI [0.04, 0.75], *p* = 0.02 (Bonferroni adj.: *p* = 0.016). A significant Emotion x Task interaction, *F*(2,118) = 3.47, *p* < 0.05, partial η^2^ = 0.06, was further explored by examining the effect of Task for each emotion separately. This showed that Task was only significant for the negative trials, for which both Emotion (*M*_diff_ = 0.59, 95% CI [0.05, 1.13], *p* = 0.03) and Content labeling (*M*_diff_ = 0.69, 95% CI [0.15, 1.23], *p* < 0.01) led to reduced arousal compared to merely viewing (**Figure 1**). No main effects or interactions with CSD scores were found for arousal ratings.

Finally, for control ratings a main effect of Emotion *F*(1,59) = 170.72, *p* < 0.001, partial η^2^ = 0.74, and an Emotion x Task interaction, *F*(2,118) = 9.87, *p* < 0.001, partial η^2^ = 0.14, showed that, as expected, negative trials led to less perceived control than positive trials, but both labeling conditions, especially content labeling (*M*_diff_ = -0.74, 95% CI [-1.17, -0.31], *p* < 0.001) resulted in an increase of perceived control during negative trials (see **Figure 1**). No effects were found for CSD scores.

Overall, these analyses indicate that (a) cues evoked the expected emotional reactions and (b) these reactions were dampened during labeling of the pictures emotionally or non-emotionally, suggesting that the task successfully produced emotion regulatory effects.

### MAIN ANALYSES

#### Affect labeling and symptom reporting

**FIGURE 3:**
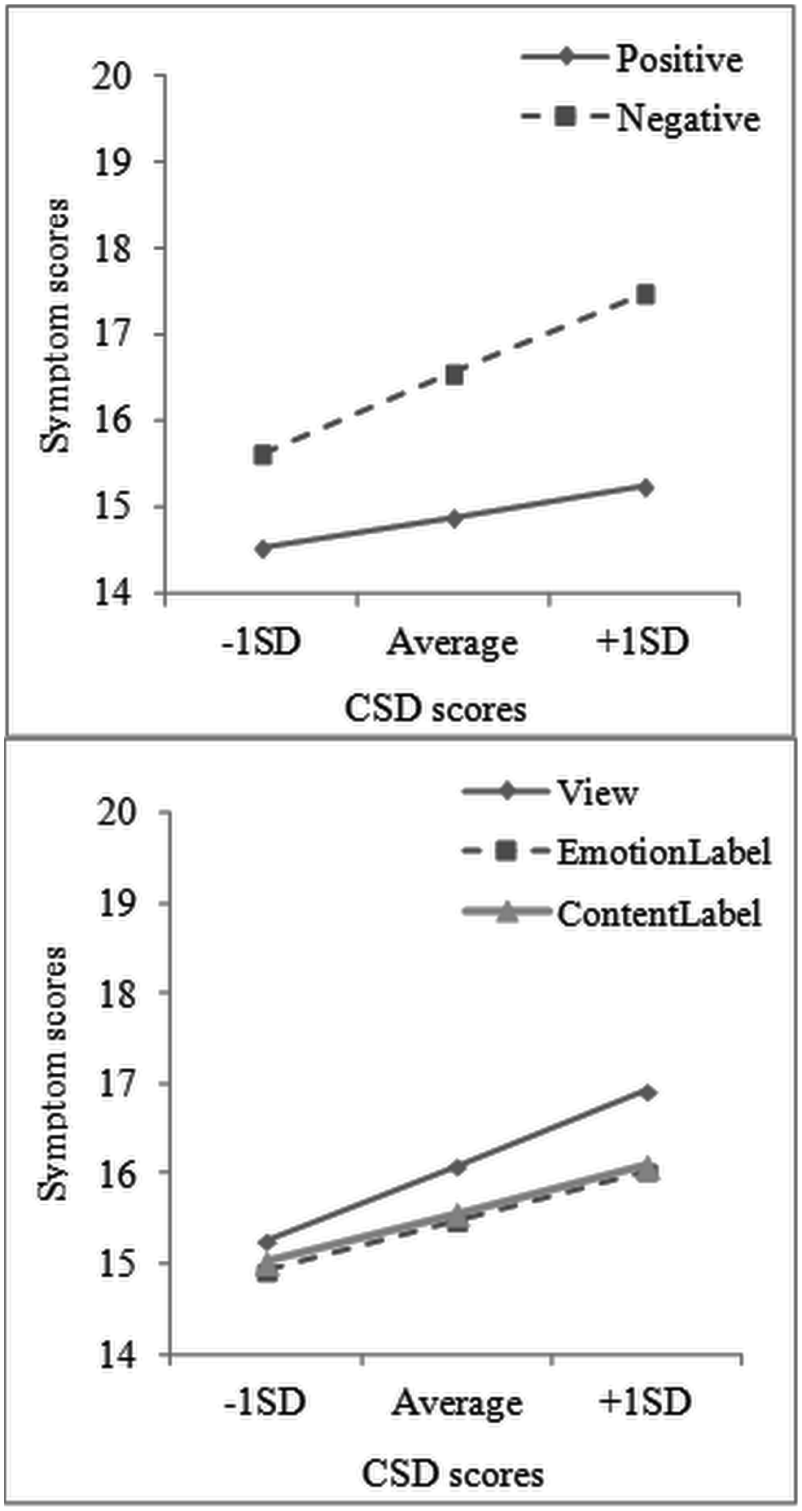
**Interaction of habitual symptom reporting scores with both Emotion (top panel) and Task (bottom panel) for symptom scores after each trial of the Affect Labeling task.** CSD = Checklist for Symptoms in Daily Life.

A repeated measures ANCOVA with the total symptom scores after each trial (see **Table 2**) as dependent variable explored labeling effects on symptom reports. First, as expected, negative trials resulted in more symptom reports compared to positive trials (Emotion main effect, *F*(1,59) = 74.30, *p* < 0.001, partial η^2^ = 0.56). Additionally, a Task main effect *F*(2,118) = 12.77, *p* < 0.001, partial η^2^ = 0.18, and an Emotion × Task, *F*(2,118) = 5.58, *p* < 0.01, partial η^2^ = 0.09, interaction were observed, which showed that both Emotion, *M*_diff_ = 1.11, 95% CI [0.53, 1.70], *p* < 0.001, and Content Labeling *M*_diff_ = 0.85, 95% CI [0.27, 1.43], *p* = 0.002, led to a reduction of symptom ratings, but only during the negative trials (**Figure 1**). As for CSD scores, these had a significant main effect on symptom reports *F*(1,59) = 12.31, *p* < 0.001, partial η^2^ = 0.17, but they also interacted significantly with both Emotion, *F*(1,59) = 8.71, *p* < 0.01, partial η^2^ = 0.13, and Task, *F*(2,118) = 3.22, *p* < 0.05, partial η^2^ = 0.05. Separate analyses showed that higher CSD scores predicted higher symptom reports at all trials, but their effect was more pronounced for negative, *F*(1,59) = 12.80, *p* < 0.001, partial η^2^ = 0.18, than positive trials, *F*(1,59) = 6.90, *p* = 0.01, partial η^2^ = 0.10, and more for view, *F*(1,59) = 14.28, *p* < 0.001, partial η^2^ = 0.19, than the two labeling conditions (*p* < 0.01 for both). Plotting the effects at different levels of CSD scores indicated that both Emotion and Task effects increased as CSD scores increased (**Figure 3**). The three-way interaction between CSD scores, Task and Emotion did not reach significance (*p* = 0.86).

#### Labeling effects on symptom reports and measures of self-regulation

To examine whether labeling effects on symptom reports relate to participants’ inhibitory capacities, we calculated difference scores subtracting the total symptom score of the viewing condition from (a) that of the Emotion Labeling condition (emotion labeling effect) and (b) that of the Content Labeling condition (content labeling effect), so that negative values represent a reduction and positive values an increase in symptom reports after labeling. As task had no effect on positive trials, only difference scores for the negative trials were calculated. Pearson’s *r* correlations indicated that the two labeling effects did not correlate significantly with the accuracy at the PGNG task (means at **Table 1**). Performance on the PGNG task did not correlate with CSD scores either. Similarly, no significant correlations were found for the HRV indices or self-reported regulatory abilities.

#### Labeling effects on symptom reports and affect ratings

**FIGURE 4:**
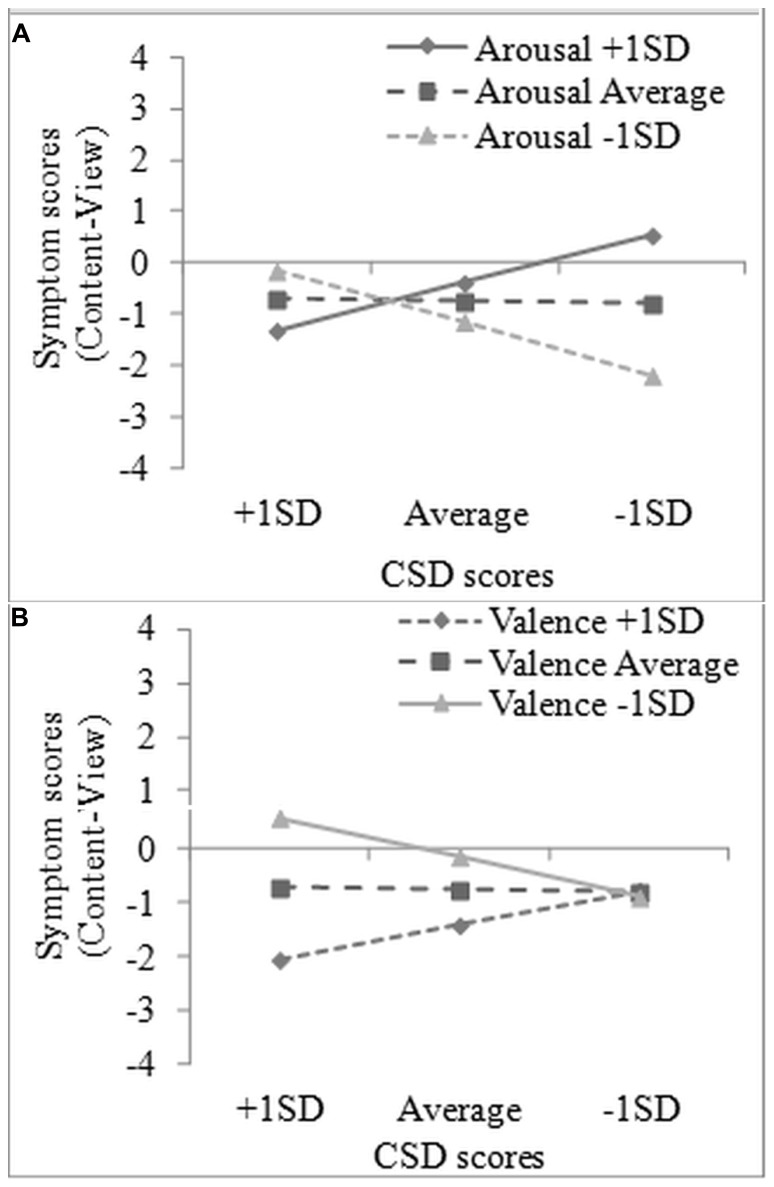
**Interaction effects predicting content labeling effect on symptom reports: (A) habitual symptom reporting × arousal ratings and (B) habitual symptom reporting × valence ratings.** CSD = Checklist for Symptoms in Daily Life.

To examine whether labeling effects on symptom reporting are predicted by the emotion regulatory effects of labeling, two multiple regressions were conducted with (a) the emotion labeling effect on symptom reports and (b) the content labeling effect on symptom reports as dependent variables. Predictors for each regression were the corresponding labeling effect on valence ratings, the corresponding labeling effect on arousal ratings, total CSD scores (all centered to the mean) and the interaction of CSD scores with valence and arousal effects. All five predictors were entered together in the regression to examine the effects of each emotional dimension (valence, arousal), while controlling for the other^3^. For the emotion labeling effect, the overall model was nearly significant, *R*^2^_adj._ = 0.10, *F*(5,55) = 2.28, *p* = 0.06. The only predictor approaching significance was arousal (β = 0.25, SE = 0.13, *t* = 1.92, *p* = 0.06), with higher reductions in arousal after Emotion Labeling predicting higher reductions in symptom reports. For the content labeling effect, the model was statistically significant, *R*^2^_adj._ = 0.12, *F*(5,55) = 2.68, *p* < 0.05. A significant interaction between CSD scores and the content labeling effect on arousal ratings was found (β = -0.60, SE = 0.24, *t* = -2.54, *p* = 0.01) and was further explored as suggested by Aiken and West (1991). Specifically, the regression slopes for three levels of CSD (average, +1SD, -1 SD) and three levels of arousal (average, +1 SD, -1 SD) were calculated and these showed that as perceived arousal decreases after Content Labeling, symptoms also decrease but only for those low in CSD (**Figure 4A**). Furthermore, the content labeling effect on valence ratings, and its interaction with CSD scores also approached significance, (β = -0.31, SE = 0.16, *t* = -1.99, *p* = 0.05 and β = -0.46, SE = 0.23, *t* = -2.00, *p* = 0.05), with increases in valence after Content Labeling resulting in a reduction of symptom reports. The interaction showed that this effect becomes more pronounced as CSD scores increase (**Figure 4B**).

## DISCUSSION

The present study investigated whether applying an implicit emotion regulation technique can reduce the well-documented augmenting effect of unpleasant cues on symptom reporting and whether individual differences in habitual symptom reporting moderate such a reduction. To this end, an affective picture paradigm previously used to induce elevated symptom reports (Bogaerts et al., 2010; Constantinou et al., 2013) was combined with an affect labeling task (Lieberman et al., 2007). Healthy participants varying in habitual symptom reporting viewed pleasant and unpleasant pictures under three conditions: passive viewing, labeling the emotion, and labeling the content of the pictures, and then completed affect ratings and a symptom checklist.

Manipulation checks indicated that labeling led to less extreme ratings of valence, arousal, and control compared to passive viewing for both pleasant and unpleasant pictures. These results are in line with studies showing that labeling can dampen both positive and negative emotions (Lieberman et al., 2011) and support its usefulness as an emotion regulation technique. Main analyses showed that labeling additionally led to a reduction in symptom reports after picture viewing. This reduction was, as expected, moderated by participants’ level of habitual symptom reporting as the difference between the two labeling conditions and passive viewing was more pronounced at higher levels of habitual symptom reporting. This suggests that people who experience frequent medically unexplained symptoms can benefit the most from labeling procedures. Regression analyses further indicated that the effects of labeling on symptom reports are predicted by its effects on experienced affect.

Interestingly, contrary to our initial hypothesis, emotional and content labeling influenced self-reported affect in a similar way, suggesting that both kinds of labeling can have emotion regulatory properties. This contradicts previous studies showing that affect labeling has a rather specific effect on inhibitory pathways in the brain, which is not found for non-emotional labeling (Lane et al., 1997; Lieberman et al., 2007). However, as Lieberman et al. (2011) pointed out, in one of the few studies examining self-reported affect, little is known about the effects of non-emotional labeling on self-reports. Current findings indicate that the specificity found for emotional labeling in brain activations, is not replicated in self-reports. This discrepancy suggests that non-emotional labeling may influence self-reported affect through different routes than those described for emotional labeling. For example, non-emotional labeling may function as distraction (as it draws attention away from the emotional components of the pictures), a strategy that seems to have comparable effects with affect labeling on self-reports (Lieberman et al., 2011) or it may be that the labeling process itself (regardless of the kind of label) in general results in attenuated affect (Lange et al., 2003; Lieberman et al., 2011). Further research is needed to delineate the mechanisms underlying the effects seen in our content labeling condition.

Besides experienced affect, both kinds of labeling also influenced symptom reporting. Symptom ratings after each trial showed that, as in previous studies (Bogaerts et al., 2010; Constantinou et al., 2013), the mere presentation of unpleasant pictures can induce elevated symptom reports. Labeling the unpleasant pictures, though, either emotionally or non-emotionally, reduced this bias, an effect that seems to be most profound in people high in habitual symptom reporting. Prior studies, as well as current findings, have shown that high symptom reporters are more prone to the influences of unpleasant cues on symptom reporting (Bogaerts et al., 2010; Constantinou et al., 2013). This has been attributed to the combination of a reduced ability to regulate emotion with more elaborate and accessible representations of symptom experiences in this selected group (Brown, 2004; Bogaerts et al., 2010). As these symptom representations are inherently linked to unpleasantness, they are assumed to be automatically triggered by affectively-congruent cues (Bower, 1981; Lang, 1995) producing the effects observed in our picture viewing paradigms.

These automatic effects of affective cues could be constrained by the activation of inhibitory control processes that regulate affect (Banich et al., 2009), which we hypothesized to be less successfully employed by high habitual symptom reporters. This assumption was supported by the finding that labeling effects were more pronounced at higher levels of habitual symptom reporting, as well as by the fact that during the view condition (where no instructions are given) higher habitual symptom reporting was related to more unpleasantness and more symptom reporting after unpleasant cues. Thus, high symptom reporters seem less able to spontaneously regulate affect, but can successfully engage in the labeling tasks and benefit more from them. These results, using a well-controlled experimental design, provide support for interventions targeting emotion regulation training, like expressive writing, in patients with functional syndromes (Broderick et al., 2005; Junghaenel et al., 2008).

The connection between emotion regulation and symptom reporting was further emphasized by regressions showing that the reduction of symptoms during labeling was predicted by reductions in experienced affect during picture viewing, especially self-reported arousal. It is important to note, though, that symptom reduction during content labeling was mostly related to arousal for low habitual symptom reporters, but to valence for high symptoms reporters. This may point to differences between the two groups in how labeling works and how symptom reports emerge. For low symptom reporters, reductions in symptoms during labeling may stem from reductions in actual physiological arousal, while for high symptom reporters labeling effects on symptom reports seem unrelated to actual bodily changes, but rather depend on the experienced unpleasantness and the resulting affectively congruent schema activations. As objective measures of autonomic arousal were not included in this study, this tentative hypothesis cannot be examined with the current data.

Another finding worth pointing out is that although emotion regulation seems to reduce affective influences on symptom reporting, especially for high habitual symptom reporters, this process does not seem to relate to their dispositional regulatory capacity. Contrary to our hypotheses, measures of general inhibitory capacity, like a motor inhibition task (PGNG task), a physiological marker (HRV) or self-reported regulatory ability, were not associated with the beneficial effects of labeling on symptom reporting nor with habitual symptom reporting. This rather surprising lack of correlations may suggest that labeling may influence symptom experiences through other processes, like distraction and people’s expectations about its effects (Lieberman et al., 2011) and not via the inhibitory effects of emotional labeling (Lieberman et al., 2007). The similar effects of emotion and content labeling on symptom reports further shows that the inhibitory processes involved in emotional labeling did not have additional effects in the context of symptom reporting.

Alternatively, this lack of correlations may also be partly due to the neutral nature of the motor inhibition task. As there is no affective-motivational component in the PGNG task, it may be less relevant to the resources required for emotion regulation (Banich et al., 2009). Although common substrates have been reported for “cold” and “hot” inhibitory control (Cohen and Lieberman, 2011), different kinds of inhibition can also have additional distinct effects (Dillon and Pizzagalli, 2007). Thus, future research using emotional equivalents of inhibitory tasks would be useful in delineating these associations. Additionally, although intentional emotion regulation has been related to motor inhibition (Tabibnia et al., 2011), implicit emotion regulation tasks may be less related to such tasks that involve intentional efforts for inhibitory control, a hypothesis that should be further explored.

Finally, several limitations of the present study should be reported. Firstly, the amount of symptoms reported by participants during the task was rather low, which is to be expected when healthy young people rate their body state without experimentally induced bodily symptoms. However, this may hamper the strength of the effects and their generalizability from mild and short-lived symptom experiences to more long-lasting, debilitating symptoms, as those experienced by patients with functional syndromes. Furthermore, current analyses used total symptom scores, thus it cannot be concluded whether the observed effects were general or specific to certain symptoms categories. Future research could try to delineate the extent of labeling effects on symptom reporting, and replicate these findings in patient populations, both in similar experimental designs, as well as in more ecologically valid paradigms (e.g., diary studies). Additionally, current results pose further questions regarding the mechanisms behind labeling effects on self-reports, which should also be addressed by future research.

## CONCLUSION

Current findings emphasize the malleability of the symptom reporting process and its influence by mild manipulations, like the presentation of unpleasant cues. This study showed that symptom reporting can easily be augmented, but also easily reduced via the application of implicit emotion regulation techniques. High habitual symptom reporters seem to be more prone to these manipulations. The study also provides first indications about the usefulness of such strategies in reducing affective biases in symptom perception, although more research is needed to verify the beneficial effects of these strategies in clinical groups (e.g., patients with functional syndromes).

## Conflict of Interest Statement

The authors declare that the research was conducted in the absence of any commercial or financial relationships that could be construed as a potential conflict of interest.

## Footnotes

1 *Positive1:* 1463, 1920, 2550, 4574, 5201, 5260, 7330, 8080, 8185, 8030; *Positive2:* 1620, 2341, 5760, 5700, 5849, 7280, 8461, 8370, 8200, 8490; *Positive 3:* 1710, 2311 2360, 5891, 8190, 7260, 8300, 8033, 8470, 8502; *Negative1:* 6242, 9001, 6190, 9911, 1525, 9410, 9425, 9426, 9520, 9561; *Negative2*: 9611, 1114, 2095, 2520, 2900, 2692, 5971, 6315, 6821, 9181; *Negative3:* 1932, 2800, 5972, 6300, 6370, 9140, 6800, 9421, 6838, 9041.

2 *Positive valence (1–9):* M1 = 7.50, SD1 = 0.36, M2 = 7.57, SD2 = 0.46, M3 = 7.55, SD3 = 0.60; *Positive arousal(1–9)*: M1 = 5.39, SD1 = 1.30, M2 = 5.09, SD2 = 1.27, M3 = 5.14, SD3 = 1.09; *Negative valence (1–9):* M1 = 2.72, SD1 = 0.79, M2 = 2.58, SD2 = 0.65, M3 = 2.72, SD3 = 0.71; *Negative arousal(1–9):* M1 = 5.80, SD1 = 0.77, M2 = 5.71, SD2 = 1.02, M3 = 5.77, SD3 = 0.75.

3 To limit the amount of predictors in the models (due to sample size restrictions), labeling effects on control ratings were not included in the regressions.
